# Supplementary material for: Coupling radiative, conductive and convective heat-transfers in a single Monte Carlo algorithm: A general theoretical framework for linear situations
Source: PLoS One. 2023 Apr 6;18(4):e0283681. doi: 10.1371/journal.pone.0283681 (PMC10079137; doi:10.1371/journal.pone.0283681)
Supplement: S1 File — (PDF) [file pone.0283681.s001.pdf]

Nomenclature

$(\vec{\boldsymbol{W}}_{\boldsymbol{\mathcal{W}}}, T_{\mathcal{W}})$ Paired random variable with distribution $p_{(\vec{\boldsymbol{W}}_{\boldsymbol{\mathcal{W}}}, T_{\mathcal{W}})} = g_{\mathcal{W}}/p_{\mathcal{W}}$

$(\vec{\boldsymbol{W}}_{\boldsymbol{\partial\mathcal{W}}}, T_{\partial\mathcal{W}})$ Paired random variable with distribution $p_{(\vec{\boldsymbol{W}}_{\boldsymbol{\partial\mathcal{W}}}, T_{\partial\mathcal{W}})} = g_{\partial\mathcal{W}}/p_{\partial\mathcal{W}}$

$(\vec{\boldsymbol{X}}_{\boldsymbol{R}}^{\boldsymbol{F_i}}, T^{F_i})$ Instantiation of $(\vec{\boldsymbol{W}}_{\boldsymbol{\mathcal{W}}}, T_{\mathcal{W}})$ in the fluid model

$(\vec{\boldsymbol{X}}_{\boldsymbol{R}}^{\boldsymbol{S}}, T^{S})$ Instantiation of $(\vec{\boldsymbol{W}}_{\boldsymbol{\mathcal{W}}}, T_{\mathcal{W}})$ in the solid model

$(\vec{\boldsymbol{Y}}_{\boldsymbol{D}}^{\boldsymbol{S}}, T^{S})$ Instantiation of $(\vec{\boldsymbol{W}}_{\boldsymbol{\partial\mathcal{W}}}, T_{\partial\mathcal{W}})$ in the solid model for the Dirichlet boundary

$(\vec{\boldsymbol{Y}}_{\boldsymbol{F}}^{\boldsymbol{S}}, T^{S})$ Instantiation of $(\vec{\boldsymbol{W}}_{\boldsymbol{\partial\mathcal{W}}}, T_{\partial\mathcal{W}})$ in the solid model for the Robin boundary

$(\vec{\boldsymbol{Y}}_{\boldsymbol{R}}^{\boldsymbol{R}}, \vec{\boldsymbol{U}}_{\boldsymbol{R}})$ Instantiation of $\vec{\boldsymbol{W}}_{\boldsymbol{\partial\mathcal{W}}}$ in the radiative transfer model

$(\vec{\boldsymbol{Y}}_{\boldsymbol{S}}^{\boldsymbol{F_i}}, T^{F_i})$ Instantiation of $(\vec{\boldsymbol{W}}_{\boldsymbol{\partial\mathcal{W}}}, T_{\partial\mathcal{W}})$ in the fluid model

α Parameter in the system of coupled functional integrals $(= \zeta/(\rho C))$

α_{F_i} Inverse characteristic time in a fluid cavity model

$\bar{h}_F$ Average convective exchange coefficient over the fluid / solid boundary

$\bar{h}_{F_i}$ Average convective exchange coefficient over the fluid / solid boundary of the ith cavity

$\bar{\Omega}_J$ The adherence of domain Ω_J

β Parameter in the system of coupled functional integrals $(= k_a c)$

$\vec{\boldsymbol{\nabla}}$ Nabla, differential operator

$\vec{\boldsymbol{\theta}}$ Vector of fluid, solid and radiative temperatures in the Fredholm equation

$\vec{\boldsymbol{f}}$ Prescribed terms in the Fredholm equation

$\vec{\boldsymbol{j}}$ Conductive energy flux density vector

$\vec{\boldsymbol{n}}$ Incoming normal vector to the solid boundary $\partial\Omega_S$

$\vec{\boldsymbol{U}}$ Random variable that follows a uniform law on the sphere

$\vec{\boldsymbol{u}}$ Direction vector

$\vec{\boldsymbol{W}}_{\boldsymbol{I}}$ Random variable with distribution $p_{\vec{\boldsymbol{W}}_{\boldsymbol{I}}} = g_I/p_I$

$\vec{\boldsymbol{X}}_{\boldsymbol{N}}$ Position random variable after N jumps of size δ

$\vec{\boldsymbol{X}}_{\boldsymbol{A}}^{\boldsymbol{R}}$ Instantiation of $\vec{\boldsymbol{W}}_{\boldsymbol{\mathcal{W}}}$ in the radiative transfer model

$\vec{\boldsymbol{x}}$ Position vector

$\vec{\boldsymbol{\mathcal{I}}}$ Linear integral vector operator acting on the temperature vector in the Fredholm equation

$\mathcal{L}$ Algebraic linear operator

$\mathcal{W}$ Generic integration domain of any dimension

$\cap$ Intersection of ensembles

$\cup$ Union of ensembles

Δ Laplacian, differential operator

δ Step of the δ-sphere random walk

$\delta(\cdot)$ Dirac distribution

δ_b Step of the finite difference discretization of the normal derivative at the solid/fluid boundary in the δ-sphere random walk

$\delta_{\partial\Omega}$ Distance to the closest boundary in the $\vec{\boldsymbol{u}}$ or $-\vec{\boldsymbol{u}}$ directions in the δ-sphere random walk

δ_{ref} Maximum step of the δ-sphere random walk

ϵ Arbitrary thickness of the boundary in the δ-sphere approximation

$\epsilon_{\mathscr{X}}$, $\epsilon_{\mathscr{R}}$ Time random variables

Γ Random variable "radiative path"

$\hat{\theta}_{F_i}$ Realization of Θ_{F_i}

$\hat{\theta}_R$ Realization of Θ_R

$\hat{\theta}_S$ Realization of Θ_S

$\hat{f}$ Realization of F

λ Thermal conductivity of the material

$\mathbb{E}$ Expectation of a random variable

$\mathbb{R}$ Real vector space of dimension 1

$\mathbb{R}^n$ Real vector space of dimension n

$\mathbb{S}^2$ 2-sphere, the ensemble of points that lie on the surface of a three-dimensional ball

$\mathbb{S}^{n-1}$ (n-1)-sphere, the ensemble of points that lie on the surface of a n-dimensional ball

$\mathbb{S}^2_+$ The ensemble of directions towards the interior of the sphere

$\mathcal{B}(p)$ Bernoulli random variable with parameter p

μ Rate at which $\vec{\mathscr{X}}$ hits the surface of the fluid domain

ν Frequency

Ω_F Fluid domain (the union of the m fluid cavities and the surrounding fluid cavity)

Ω_S Solid domain

$\Omega_{F\infty}$ Surrounding fluid cavity

Ω_{F_i} i^{th} fluid cavity

$\mathring{\Omega}_J$ The interior of domain Ω_J

∂n Partial derivative with respect to n

$\partial\Omega_D$ Part of $\partial\Omega_S$ with Dirichlet-type boundary conditions

$\partial\Omega_F$ Boundary of the union of fluid cavities

$\partial\Omega_R$ Fictitious spherical boundary enclosing the whole domain

$\partial\Omega_S$ Boundary of the (disconnected) solid medium

$\partial\Omega_{F_i}$ Boundary of the ith fluid cavity

$\partial\Omega_J^K$ The complementary of $\partial\Omega_J$ to $\partial\Omega_K$

∂_θ Partial derivative with respect to temperature

∂_t Partial derivative with respect to time

$\partial\mathcal{W}$ Boundary of the generic integration domain

ψ_R Radiative power density, difference between absorbed and emitted power densities

ρ Mass density of the material

ρ_i Mass density of the fluid in the ith cavity

$\tau_{\mathscr{X}}$, $\tau_{\mathscr{R}}$ Random variables, the time at which the associated process reaches the parabolic boundary

θ Temperature

θ_1 Observable

θ_2 Function of a random variable X

θ_D Temperature on $\partial\Omega_D$

θ_F Temperature in the fluid

θ_I Initial temperature at time t_I

θ_R Radiative temperature (the angular and spectral integral of the monochromatice radiance temperature)

θ_S Temperature in the solid

$\theta_{F/S}$ Temperature in the fluid or solid depending on location

θ_{F^∞} Temperature in the surrounding fluid cavity

θ_{F_i} Temperature in ith fluid cavity

$\Theta_{R,\vec{\boldsymbol{U}}}$ Random variable whose expectation is θ_R

$\Theta_{R,\vec{\boldsymbol{u}}}$ Random variable whose expectation is $\theta_{R,\vec{\boldsymbol{u}}}$

$\theta_{R,\vec{\boldsymbol{u}}}$ Radiance temperature (or brightness temperature) associated with the monochromatic specific intensity of frequency ν in direction $\vec{\boldsymbol{u}}$

$\theta^\nu_{R,\vec{\boldsymbol{u}}}$ Monochromatic radiance temperature (or brightness temperature) associated with the monochromatic specific intensity of frequency ν in direction $\vec{\boldsymbol{u}}$

θ_{ref} Reference temperature for linearized radiative transfer

Θ_S Random variable whose expectation is θ_S

$\tilde{I}_\nu$ Monochromatic specific intensity perturbation with respect to equilibrium

$\tilde{\theta}_R, \theta_{R,\partial\Omega_R,\vec{\boldsymbol{u}}}$ Radiance temperature on $\partial\Omega_R$

$\tilde{\Theta}_{F_i}$, Θ_{F_i} Random variable whose expectation is θ_{F_i}

$\tilde{\Theta}_{int}$ Random variable whose expectation is $\tilde{\theta}_S$

$\tilde{\theta}_{S,\vec{\boldsymbol{u}}}$ The temperature of the solid in the δ-sphere approximation, at $(\vec{\boldsymbol{x}} + \delta\vec{\boldsymbol{u}}, t)$

$\tilde{\theta}_S$ The temperature of the solid in the δ-sphere approximation, at $(\vec{\boldsymbol{x}}, t)$

$\vec{\boldsymbol{w}}$ Point in $\mathcal{W}$

$\vec{\mathscr{X}}$, $\vec{\mathscr{R}}$, $\vec{\mathscr{U}}$ Stochastic processes defined on either a solid or fluid domain, on the whole domain (union of solid and fluid cavities) and on the unit sphere respectively

$\vec{\mathscr{X}}_p^{\vec{\boldsymbol{x}},t}$ $\vec{\mathscr{X}}$ at time p conditioned to reach $\vec{\boldsymbol{x}}$ at time t

ζ Linearized radiative transfer coefficient in the solid

ζ_i Linearized radiative transfer coefficient in the ith fluid cavity

A Random variable whose expectation is θ_2

a Realization of A

$a(\vec{\boldsymbol{w}}, t)$, $c(\vec{\boldsymbol{w}}, t)$ Functions that are part of the generic model definition

b Realization of B

B, $\tilde{B}$ Random variables whose expectation is θ_1

C Heat capacity of the material

C_i Heat capacity of the fluid in the ith cavity

D Thermal diffusivity of the material in the δ-sphere approximation

F Random variable whose expectation is f

f Generic quantity of interest (real-valued function)

$f_{\mathcal{W}}$, $f_{\partial\mathcal{W}}$, f_I Generic source terms (real-valued functions)

g The Green function in the generic model

g_R The Green function for the radiative transfer model

g_S The Green function in the solid model

$g_{\mathcal{W}}$, $g_{\partial\mathcal{W}}$, g_I Propagators of the generic source terms

$g_{F_i,I}$, $g_{F_i,S}$, $g_{F_i,R}$ Instantiation of the propagators of the source terms in the fluid model (initial conditions, solid boundary, radiation in the volume)

$g_{R,\partial\Omega_R}$, $g_{R,A}$ Instantiation of the propagators of the source terms in the radiative transfer model (boundary conditions, absorption/emission in the volume)

$g_{S,I}$, $g_{S,\partial\Omega_D}$, $g_{S,\partial\Omega_F^p}$, $g_{S,R}$ Instantiation of the propagators of the source terms in the solid model (initial conditions, Dirichlet and Robin boundary conditions, radiation in the volume)

h_F Convective exchange coefficient at the fluid / solid boundary

$H_J(k)$ Test function, 1 if $k \in J$, 0 otherwise

I_ν Monochromatic specific intensity

I_ν^{eq} Monochromatic specific equilibrium intensity

k_a^ν Absorption coefficient at frequency ν

k_e^ν Extinction coefficient at frequency ν

k_s^ν Scattering coefficient at frequency ν

L, $L_{\partial\mathcal{W}}$ Generic homogeneous and linear integrodifferential operators

l_p Local boundary time process

m Number of fluid cavities

n Dimension of the geometric space in the δ-sphere approximation

p_A^R For radiative model : probability of ending at a solid ou fluid temperature

p_I, $p_{\partial\mathcal{W}}$, $p_{\mathcal{W}}$ Probabilities associated with the different sources

$p_I^{F_i}$, $p_R^{F_i}$ For fluid model : probability of ending at initial condition, probability of ending at radiative temperature

p_I^S, p_2^S, p_3^S For solid model : probability of ending at initial condition, probability of ending at a radiative temperature, probablity of endind at a fluid temperature

p_N Frequency probability distribution function

p_S^ν Phase function at frequency ν

$p_X(x)$ Probability density function associated with the random variable X

q_F Either θ_I or θ_D

q_S Either θ_I, θ_F or θ_D

r_F Either θ_I, θ_S or θ_R

r_R Either $\tilde{\theta}_R$, θ_S or θ_F

r_S Either θ_I, θ_D, θ_F or θ_R

T Random variable "time"

t Time

t_I Time of the initial condition

W Three-dimensional Brownian motion

X Random variable

x Realization of X

$\mathcal{D}_\Gamma$ Radiative path space

$\mathcal{H}(x)$ Heaviside function, 1 if $x > 0$, 0 otherwise

$\mathcal{S}_F$ Area of the boundary of the union of fluid cavities $\partial\Omega_F$

$\mathcal{V}_{F_i}$ Volume of the ith fluid cavity

MC Monte Carlo

RTE Radiative Transfer Equation

WoS Walk on Sphere
