# Supplementary material for: Coupling radiative, conductive and convective heat-transfers in a single Monte Carlo algorithm: A general theoretical framework for linear situations
Source: PLoS One. 2023 Apr 6;18(4):e0283681. doi: 10.1371/journal.pone.0283681 (PMC10079137; doi:10.1371/journal.pone.0283681)
Supplement: Attachment — Submitted filename: PLOSONE Recommendation Report and Questions.docx [file pone.0283681.s002.docx]

The authors combine the radiation, convection, and conduction phenomenon using the single Monte Carlo algorithm. The algorithm would be beneficial for the complex geometries from computer graphics and its advances. The theoretical formulation has been presented and different thermal paths have been made on which heat is transferred. They probabilized Green’s function and propagators using a theoretical framework. Then they extended the model to make it operational by using the Feynman-Kac theory and stochastic processes. They presented Brownian trajectories and make them compatible with the algorithmic design required by the ray-tracing acceleration technique for highly refined geometries.

Following are my suggestion for this manuscript:

1) In different section of the article, the flow chart the sample computer code is written. I think there is a need of a complete flow chart that describe the algorthim start and end points by highlighting the coupling spotlights. The flow chart can also describe fluid and solid domains for heating pathways.
2) I think there should be a portion that describe that why this type of coupling is required for a physical phenomenon and what kind of target audience would be beneficial.
3) The introduction section is very brief also not including the work of other people. How the people have worked previously. How thery tried to couple this type of models. Research gap needs to be highlighted.
4) The randomized sampling method should be explain more with kind of error analysis. So the path for next step should be determined correctly. The stepping method and size for the path needs more mathematical explanation.
5) In figure 7b, the radiative path is making a triangle, is there a specific reason for the twist or sampling method is changing it? More explanation is required for changing path.
6) Figure 7b can be made separately with an enlarge view.
7) Equation 57 is an average of quantities. Why is it so? The exactness of this relation can be highlighted mathematically.
